# Supplementary material for: Modeling glioblastoma heterogeneity as a dynamic network of cell states
Source: Mol Syst Biol. 2021 Sep 16;17(9):e10105. doi: 10.15252/msb.202010105 (PMC8444284; doi:10.15252/msb.202010105)
Supplement: Supplementary file 6 — Source Data for Figure 5 [file MSB-17-e10105-s004.zip › Figure5A_sourcedata/GSEA_3017/nosigdb_stateF.GseaPreranked.1622795015644/RICHARDS_NOWAKOWSKI_SCIENCE_2017_VRG_UPREG.html]

Details for gene set RICHARDS\_NOWAKOWSKI\_SCIENCE\_2017\_VRG\_UPREG[GSEA]

|  || Dataset | state33017 |
| Phenotype | NoPhenotypeAvailable |
| Upregulated in class | na\_pos |
| GeneSet | RICHARDS\_NOWAKOWSKI\_SCIENCE\_2017\_VRG\_UPREG |
| Enrichment Score (ES) | 0.40988043 |
| Normalized Enrichment Score (NES) | 2.8582306 |
| Nominal p-value | 0.0 |
| FDR q-value | 0.0 |
| FWER p-Value | 0.0 |
Table: GSEA Results Summary

  

Fig 1: Enrichment plot: RICHARDS\_NOWAKOWSKI\_SCIENCE\_2017\_VRG\_UPREG      
 Profile of the Running ES Score & Positions of GeneSet Members on the Rank Ordered List

  

| PROBE | GENE SYMBOL | GENE\_TITLE | RANK IN GENE LIST | RANK METRIC SCORE | RUNNING ES | CORE ENRICHMENT || 1 | CLU |  |  | 5 | 1.852 | 0.0190 | Yes |
| 2 | ATP1B2 |  |  | 9 | 1.759 | 0.0378 | Yes |
| 3 | HOPX |  |  | 14 | 1.345 | 0.0514 | Yes |
| 4 | CST3 |  |  | 15 | 1.273 | 0.0661 | Yes |
| 5 | LGALS3 |  |  | 16 | 1.261 | 0.0806 | Yes |
| 6 | SLC1A3 |  |  | 26 | 1.058 | 0.0885 | Yes |
| 7 | SDCBP |  |  | 27 | 1.054 | 0.1007 | Yes |
| 8 | LRRC17 |  |  | 31 | 1.044 | 0.1113 | Yes |
| 9 | RPS27L |  |  | 33 | 1.025 | 0.1226 | Yes |
| 10 | DKK3 |  |  | 37 | 0.994 | 0.1326 | Yes |
| 11 | IFITM3 |  |  | 38 | 0.987 | 0.1440 | Yes |
| 12 | F3 |  |  | 41 | 0.978 | 0.1543 | Yes |
| 13 | SAT1 |  |  | 45 | 0.957 | 0.1639 | Yes |
| 14 | GFAP |  |  | 47 | 0.953 | 0.1744 | Yes |
| 15 | MLC1 |  |  | 58 | 0.896 | 0.1800 | Yes |
| 16 | MYO6 |  |  | 62 | 0.859 | 0.1884 | Yes |
| 17 | SLC1A2 |  |  | 63 | 0.857 | 0.1983 | Yes |
| 18 | SRGAP3 |  |  | 66 | 0.850 | 0.2072 | Yes |
| 19 | COL4A5 |  |  | 71 | 0.829 | 0.2148 | Yes |
| 20 | PON2 |  |  | 93 | 0.764 | 0.2135 | Yes |
| 21 | LTBP1 |  |  | 108 | 0.741 | 0.2153 | Yes |
| 22 | NCAN |  |  | 138 | 0.686 | 0.2092 | Yes |
| 23 | PTGFRN |  |  | 151 | 0.664 | 0.2110 | Yes |
| 24 | TTYH3 |  |  | 154 | 0.655 | 0.2176 | Yes |
| 25 | FAM84A |  |  | 157 | 0.650 | 0.2242 | Yes |
| 26 | DDR1 |  |  | 159 | 0.649 | 0.2312 | Yes |
| 27 | ITM2C |  |  | 160 | 0.649 | 0.2387 | Yes |
| 28 | APC |  |  | 169 | 0.632 | 0.2421 | Yes |
| 29 | DTNA |  |  | 176 | 0.617 | 0.2463 | Yes |
| 30 | FUT8 |  |  | 194 | 0.593 | 0.2450 | Yes |
| 31 | C1orf61 |  |  | 202 | 0.587 | 0.2484 | Yes |
| 32 | HIGD1A |  |  | 212 | 0.580 | 0.2507 | Yes |
| 33 | HSPA4L |  |  | 215 | 0.579 | 0.2564 | Yes |
| 34 | PDGFC |  |  | 219 | 0.577 | 0.2616 | Yes |
| 35 | PDLIM5 |  |  | 223 | 0.572 | 0.2668 | Yes |
| 36 | HTRA1 |  |  | 229 | 0.568 | 0.2709 | Yes |
| 37 | SCHIP1 |  |  | 233 | 0.566 | 0.2760 | Yes |
| 38 | SRGAP2C |  |  | 238 | 0.563 | 0.2806 | Yes |
| 39 | LIFR |  |  | 239 | 0.562 | 0.2871 | Yes |
| 40 | MEGF10 |  |  | 240 | 0.560 | 0.2935 | Yes |
| 41 | PHYHIPL |  |  | 245 | 0.557 | 0.2980 | Yes |
| 42 | ARL6IP5 |  |  | 264 | 0.542 | 0.2956 | Yes |
| 43 | HMGCS1 |  |  | 271 | 0.537 | 0.2989 | Yes |
| 44 | SPAG16 |  |  | 273 | 0.535 | 0.3046 | Yes |
| 45 | FAM19A5 |  |  | 277 | 0.527 | 0.3092 | Yes |
| 46 | BCAN |  |  | 302 | 0.510 | 0.3035 | Yes |
| 47 | PTPRZ1 |  |  | 310 | 0.505 | 0.3059 | Yes |
| 48 | TMEM47 |  |  | 316 | 0.502 | 0.3093 | Yes |
| 49 | HES1 |  |  | 320 | 0.501 | 0.3137 | Yes |
| 50 | IL6ST |  |  | 321 | 0.500 | 0.3194 | Yes |
| 51 | CNTNAP3B |  |  | 324 | 0.498 | 0.3242 | Yes |
| 52 | PSAT1 |  |  | 329 | 0.496 | 0.3280 | Yes |
| 53 | SEMA5A |  |  | 336 | 0.494 | 0.3308 | Yes |
| 54 | ITGAV |  |  | 344 | 0.490 | 0.3331 | Yes |
| 55 | SYT11 |  |  | 352 | 0.486 | 0.3353 | Yes |
| 56 | ATP2B4 |  |  | 356 | 0.485 | 0.3394 | Yes |
| 57 | PDCD4 |  |  | 360 | 0.484 | 0.3436 | Yes |
| 58 | ABAT |  |  | 362 | 0.483 | 0.3487 | Yes |
| 59 | GATM |  |  | 375 | 0.480 | 0.3484 | Yes |
| 60 | ALDOA |  |  | 377 | 0.479 | 0.3535 | Yes |
| 61 | SEMA6A |  |  | 381 | 0.478 | 0.3575 | Yes |
| 62 | METTL7A |  |  | 403 | 0.469 | 0.3528 | Yes |
| 63 | VCAN |  |  | 410 | 0.466 | 0.3553 | Yes |
| 64 | PSAP |  |  | 418 | 0.463 | 0.3572 | Yes |
| 65 | FAM171B |  |  | 422 | 0.462 | 0.3611 | Yes |
| 66 | NADK2 |  |  | 437 | 0.456 | 0.3596 | Yes |
| 67 | TRPS1 |  |  | 441 | 0.455 | 0.3634 | Yes |
| 68 | MAST4 |  |  | 446 | 0.451 | 0.3667 | Yes |
| 69 | TTYH1 |  |  | 457 | 0.448 | 0.3670 | Yes |
| 70 | ATXN2 |  |  | 477 | 0.442 | 0.3630 | Yes |
| 71 | JUND |  |  | 478 | 0.442 | 0.3680 | Yes |
| 72 | RFTN2 |  |  | 485 | 0.439 | 0.3702 | Yes |
| 73 | LPIN2 |  |  | 488 | 0.438 | 0.3743 | Yes |
| 74 | HEATR5A |  |  | 491 | 0.438 | 0.3784 | Yes |
| 75 | NDUFB5 |  |  | 498 | 0.435 | 0.3805 | Yes |
| 76 | YBX3 |  |  | 509 | 0.431 | 0.3807 | Yes |
| 77 | PEA15 |  |  | 521 | 0.427 | 0.3803 | Yes |
| 78 | CA12 |  |  | 522 | 0.426 | 0.3852 | Yes |
| 79 | PELI2 |  |  | 529 | 0.422 | 0.3872 | Yes |
| 80 | TFPI |  |  | 531 | 0.421 | 0.3916 | Yes |
| 81 | FKBP9 |  |  | 533 | 0.419 | 0.3959 | Yes |
| 82 | MOXD1 |  |  | 549 | 0.415 | 0.3934 | Yes |
| 83 | GPM6B |  |  | 553 | 0.413 | 0.3968 | Yes |
| 84 | BICD1 |  |  | 563 | 0.410 | 0.3972 | Yes |
| 85 | GRB14 |  |  | 566 | 0.409 | 0.4009 | Yes |
| 86 | CNN3 |  |  | 576 | 0.407 | 0.4012 | Yes |
| 87 | RAB31 |  |  | 580 | 0.404 | 0.4045 | Yes |
| 88 | FOS |  |  | 592 | 0.401 | 0.4038 | Yes |
| 89 | ARAP2 |  |  | 599 | 0.399 | 0.4055 | Yes |
| 90 | TNC |  |  | 613 | 0.393 | 0.4037 | Yes |
| 91 | REXO2 |  |  | 620 | 0.392 | 0.4054 | Yes |
| 92 | NTRK3 |  |  | 621 | 0.391 | 0.4099 | Yes |
| 93 | ID4 |  |  | 659 | 0.383 | 0.3964 | No |
| 94 | NFE2L2 |  |  | 663 | 0.381 | 0.3994 | No |
| 95 | RGS16 |  |  | 691 | 0.373 | 0.3906 | No |
| 96 | SH3BGRL |  |  | 695 | 0.373 | 0.3935 | No |
| 97 | FABP7 |  |  | 701 | 0.371 | 0.3954 | No |
| 98 | SIPA1L1 |  |  | 717 | 0.367 | 0.3924 | No |
| 99 | FAM107A |  |  | 718 | 0.367 | 0.3966 | No |
| 100 | COL27A1 |  |  | 760 | 0.356 | 0.3809 | No |
| 101 | FLCN |  |  | 772 | 0.353 | 0.3797 | No |
| 102 | LRP4 |  |  | 773 | 0.353 | 0.3837 | No |
| 103 | FSTL1 |  |  | 774 | 0.352 | 0.3878 | No |
| 104 | MSI2 |  |  | 780 | 0.352 | 0.3895 | No |
| 105 | SLC12A2 |  |  | 782 | 0.351 | 0.3930 | No |
| 106 | FBXO32 |  |  | 800 | 0.347 | 0.3888 | No |
| 107 | RGL1 |  |  | 831 | 0.341 | 0.3783 | No |
| 108 | DAG1 |  |  | 834 | 0.340 | 0.3812 | No |
| 109 | TSPAN6 |  |  | 838 | 0.340 | 0.3837 | No |
| 110 | RHOQ |  |  | 843 | 0.339 | 0.3857 | No |
| 111 | RCN1 |  |  | 849 | 0.337 | 0.3872 | No |
| 112 | ZFP36L2 |  |  | 850 | 0.336 | 0.3910 | No |
| 113 | MSMO1 |  |  | 860 | 0.333 | 0.3905 | No |
| 114 | RHOJ |  |  | 874 | 0.331 | 0.3881 | No |
| 115 | ZFHX4 |  |  | 878 | 0.330 | 0.3904 | No |
| 116 | STAT3 |  |  | 880 | 0.330 | 0.3937 | No |
| 117 | SCRN1 |  |  | 904 | 0.325 | 0.3864 | No |
| 118 | IGDCC4 |  |  | 911 | 0.324 | 0.3872 | No |
| 119 | NCAM2 |  |  | 915 | 0.324 | 0.3895 | No |
| 120 | PDCD6 |  |  | 917 | 0.323 | 0.3928 | No |
| 121 | IFI6 |  |  | 922 | 0.323 | 0.3946 | No |
| 122 | ATP10D |  |  | 938 | 0.320 | 0.3910 | No |
| 123 | TLN2 |  |  | 944 | 0.318 | 0.3923 | No |
| 124 | GAB1 |  |  | 958 | 0.316 | 0.3896 | No |
| 125 | SLITRK2 |  |  | 967 | 0.315 | 0.3894 | No |
| 126 | FAT1 |  |  | 968 | 0.315 | 0.3930 | No |
| 127 | ITGB8 |  |  | 970 | 0.314 | 0.3962 | No |
| 128 | ZMAT1 |  |  | 983 | 0.313 | 0.3940 | No |
| 129 | ALDH6A1 |  |  | 987 | 0.311 | 0.3962 | No |
| 130 | EVI5 |  |  | 994 | 0.310 | 0.3968 | No |
| 131 | EMC2 |  |  | 1001 | 0.310 | 0.3975 | No |
| 132 | PCMTD1 |  |  | 1026 | 0.305 | 0.3894 | No |
| 133 | PCGF5 |  |  | 1045 | 0.301 | 0.3842 | No |
| 134 | MSI1 |  |  | 1051 | 0.300 | 0.3853 | No |
| 135 | NRBP2 |  |  | 1060 | 0.298 | 0.3848 | No |
| 136 | TSPAN3 |  |  | 1071 | 0.297 | 0.3834 | No |
| 137 | AEBP2 |  |  | 1090 | 0.294 | 0.3781 | No |
| 138 | SEMA5B |  |  | 1147 | 0.285 | 0.3544 | No |
| 139 | DMD |  |  | 1153 | 0.285 | 0.3553 | No |
| 140 | STXBP3 |  |  | 1164 | 0.283 | 0.3537 | No |
| 141 | LAMA1 |  |  | 1173 | 0.283 | 0.3531 | No |
| 142 | ZBTB20 |  |  | 1181 | 0.281 | 0.3530 | No |
| 143 | KCNN3 |  |  | 1182 | 0.281 | 0.3562 | No |
| 144 | CTNNB1 |  |  | 1190 | 0.280 | 0.3561 | No |
| 145 | DOK5 |  |  | 1193 | 0.279 | 0.3583 | No |
| 146 | CKB |  |  | 1200 | 0.279 | 0.3587 | No |
| 147 | JAM2 |  |  | 1204 | 0.279 | 0.3604 | No |
| 148 | FZD3 |  |  | 1214 | 0.277 | 0.3593 | No |
| 149 | CTNNA1 |  |  | 1228 | 0.276 | 0.3562 | No |
| 150 | MAPK1 |  |  | 1250 | 0.274 | 0.3492 | No |
| 151 | CHP1 |  |  | 1251 | 0.274 | 0.3524 | No |
| 152 | NBPF14 |  |  | 1258 | 0.273 | 0.3526 | No |
| 153 | SALL2 |  |  | 1274 | 0.271 | 0.3485 | No |
| 154 | KIF1B |  |  | 1276 | 0.271 | 0.3511 | No |
| 155 | NCKAP5 |  |  | 1279 | 0.271 | 0.3533 | No |
| 156 | IRF2BP2 |  |  | 1311 | 0.267 | 0.3414 | No |
| 157 | RFX4 |  |  | 1320 | 0.266 | 0.3406 | No |
| 158 | GLUD1 |  |  | 1334 | 0.264 | 0.3374 | No |
| 159 | ZDHHC2 |  |  | 1342 | 0.262 | 0.3370 | No |
| 160 | SLCO1C1 |  |  | 1372 | 0.260 | 0.3260 | No |
| 161 | ABCD3 |  |  | 1381 | 0.259 | 0.3252 | No |
| 162 | ITGA2 |  |  | 1395 | 0.257 | 0.3218 | No |
| 163 | EEPD1 |  |  | 1397 | 0.257 | 0.3243 | No |
| 164 | MEIS1 |  |  | 1407 | 0.256 | 0.3229 | No |
| 165 | NOTCH2 |  |  | 1420 | 0.255 | 0.3201 | No |
| 166 | PTAR1 |  |  | 1441 | 0.254 | 0.3133 | No |
| 167 | CUL3 |  |  | 1483 | -0.253 | 0.2965 | No |
| 168 | HSPH1 |  |  | 1495 | -0.258 | 0.2941 | No |
| 169 | ANXA2 |  |  | 1511 | -0.263 | 0.2899 | No |
| 170 | CREB5 |  |  | 1561 | -0.275 | 0.2694 | No |
| 171 | ZFP36L1 |  |  | 1590 | -0.284 | 0.2592 | No |
| 172 | LHX2 |  |  | 1597 | -0.285 | 0.2596 | No |
| 173 | ENO1 |  |  | 1673 | -0.308 | 0.2269 | No |
| 174 | RDX |  |  | 1678 | -0.310 | 0.2286 | No |
| 175 | PHLDA1 |  |  | 1718 | -0.324 | 0.2135 | No |
| 176 | GLO1 |  |  | 1755 | -0.342 | 0.2000 | No |
| 177 | NES |  |  | 1811 | -0.370 | 0.1778 | No |
| 178 | BARD1 |  |  | 1839 | -0.384 | 0.1692 | No |
| 179 | SQLE |  |  | 1863 | -0.396 | 0.1626 | No |
| 180 | PHIP |  |  | 1992 | -0.483 | 0.1064 | No |
| 181 | CACYBP |  |  | 2028 | -0.518 | 0.0955 | No |
| 182 | FABP5 |  |  | 2033 | -0.523 | 0.0996 | No |
| 183 | PPA1 |  |  | 2049 | -0.534 | 0.0985 | No |
Table: GSEA details [plain text format]

  

Fig 2: RICHARDS\_NOWAKOWSKI\_SCIENCE\_2017\_VRG\_UPREG: Random ES distribution      
 Gene set null distribution of ES for **RICHARDS\_NOWAKOWSKI\_SCIENCE\_2017\_VRG\_UPREG**
